# Childhood brain tumour risk and its association with wireless phones: a commentary

**DOI:** 10.1186/1476-069X-10-106

**Published:** 2011-12-19

**Authors:** Fredrik Söderqvist, Michael Carlberg, Kjell Hansson Mild, Lennart Hardell

**Affiliations:** 1 Department of Oncology, University Hospital, SE-701 82 Örebro, Sweden; 2 Department of Radiation Sciences, Umeå University, SE-901 87 Umeå, Sweden

**Keywords:** mobile phone, cordless phone, DECT, glioma, astrocytoma, adolescence, radiofrequency electromagnetic fields, CEFALO

## Abstract

Case-control studies on adults point to an increased risk of brain tumours (glioma and acoustic neuroma) associated with the long-term use of mobile phones. Recently, the first study on mobile phone use and the risk of brain tumours in children and adolescents, CEFALO, was published. It has been claimed that this relatively small study yielded reassuring results of no increased risk. We do not agree. We consider that the data contain several indications of increased risk, despite low exposure, short latency period, and limitations in the study design, analyses and interpretation. The information certainly cannot be used as reassuring evidence against an association, for reasons that we discuss in this commentary.

## Background

There has been a rapid increase in the use of mobile phones in recent years. Today about 5 billion people worldwide are estimated to be users, many of whom are children and teenagers. Other wireless phones, e.g. cordless phones of the Digital Enhanced Cordless Telecommunication (DECT) type, are also common and can be as frequently used, if not more so. Both give a relatively high exposure to radiofrequency electromagnetic fields (RF-EMF) [1], an environmental exposure that the International Agency for Research on Cancer (IARC) has recently classified as 'possibly carcinogenic to humans', Group 2B [2].

Meta-analyses have shown an increased risk of malignant brain tumours and acoustic neuroma associated with long-term (> 10 years) use of mobile phone, and particularly where laterality has been considered, i.e., a tumour has developed on the same side of the head as the phone was generally used [3-6]. The Hardell group also analysed the risk in different age categories for first use of mobile or cordless phones [3,7]. First use before the age of 20 yielded the highest risk, as did use in the youngest age group at diagnosis [8]. This higher risk associated with use at a young age may reflect potentially higher susceptibility to RF-EMF among children and adolescents [9]. The near field exposure to RF-EMF from a handset to the brain means that the absorbed energy may be higher for young persons than adults [10] due to their thinner bones, smaller heads and higher conductivity of their brains. The developing brain is also more sensitive than an adult brain to toxins [9]. Despite these considerations data on children are scarce.

The first study on mobile phone use and the risk of brain tumours in children and adolescents, CEFALO, has recently been published [11]. In this commentary, we will discuss its design, findings and, importantly, the authors' conclusions since this issue is highly relevant to public health [A].

### CEFALO

The multi-centre case-control study, CEFALO, conducted in Denmark, Sweden, Norway, and Switzerland, included children and adolescents aged 7-19 years diagnosed with a brain tumour between 2004 and 2008 [11]. The results were based on interviews with 352 cases (83.2% participated) and 646 controls (71.1% participated) and their parents. Controls were selected randomly, mostly from population registries [B], and matched by age, sex and geographical region. The study period was from January 1, 2004 until August 31, 2008, but varied "slightly between study centres". Conditional logistic regression models were used for statistical analysis. The reference category in the main analyses for calculation of odds ratios (OR) and corresponding 95% confidence intervals (CI) consisted of subjects who were either non-users or non-regular users of mobile phones. The latter were those who reported < 1 call per week for at least 6 months.

In the summary the authors state that they "did not observe that regular use of a mobile phone increased the risk of brain tumors". This conclusion was accompanied by an editorial stating that the study showed "no increased risk of brain tumors" [12], and a news release from the Karolinska Institute in Stockholm that the results were "reassuring" of no increased risk [13]. We do not agree with these conclusions, and will address different issues in the study to explain why this is the case.

## Discussion

### Risk related to mobile phone use

Regular use of mobile phones was reported by 55% (n = 194) of the cases and 51% (n = 329) of the controls. The study yielded a statistically non-significant, but increased risk for brain tumours among regular users of mobile phones (OR 1.36; 95% CI 0.92 to 2.02). However, if the study had been implemented as designed [14], with 550 cases and 2 controls per case, it would have been able to detect a 35% increased risk with 80% power. In other words, if the investigators had had 550 cases in the study and the overall result was similar, the 36% increased risk in brain tumours reported for 'regular' mobile phone users could have been statistically significant. With 352 cases, the study could only detect a 45% increased risk as statistically significant with the same (80%) power.

Nevertheless, in the absence of a statistically significant trend, the OR in question increased with cumulative duration of subscriptions and duration of calls. At most only a latency time of > 5 years reported by 46 cases and 81 controls was presented, with no data on longer-term use. In fact, the numbers of cases and controls is unclear, since the report states that for a latency time of 5 years, 33 cases and 45 controls were exposed-numbers that are not compatible with those for > 5 years.

The authors also present results of stratified analyses that yielded ORs ranging from 1.49 to 1.73 for Denmark, Sweden and Switzerland. In contrast, Norway's OR was 0.51, although "...in line with random variability (P for heterogeneity = 0.20)" as noted by the authors without further discussion. If the ORs 1.73, 1.49, 1.69 and 0.51 are found by the test to be 'homogeneous', then the power of the test should be questioned. The latter and the clearly lower response rate for Norway than for the other 3 countries give reason to worry that this may indicate some important methodological difference or bias.

Further support of a true association was found in the results based on operator-recorded use for 62 cases and 101 controls, which for time since first subscription > 2.8 years yielded OR 2.15 (95% CI 1.07-4.29) with a statistically significant trend (P = 0.001). The results based on such records would be judged to be more objective than face-to-face interviews, as in the study, that clearly disclosed to the interviewer who was a case or a control. The authors disregarded these results on the grounds that there was no significant trend for operator data for the other variables -- cumulative duration of subscriptions, cumulative duration of calls and cumulative number of calls. However, the statistical power in all the latter groups was lower since data was missing for about half of the cases and controls with operator-recorded use, which could very well explain the difference in the results.

### Risk related to localisation, tumour morphology and laterality

An increased risk was found for localisations of brain tumours other than the most exposed, e.g. temporal lobe, frontal lobe and cerebellum, which of course present a problem for drawing causal conclusions. Indeed, this was one of the authors' 2 main arguments as to why they thought the relationship was not causal. Instead, the authors argue that prodromal symptoms before diagnosis may have prompted the use of mobile phones and thereby possibly introduced reverse causality. However, this explanation seems unfounded for 2 reasons. First, to our knowledge there are no studies where this hypothesis gains empirical support. Second, the severity and duration of prodromal symptoms are highly dependent on type of brain tumour; hence, such symptoms are unlikely to have occurred for long enough for the vast majority of the duration of exposure in most patients to explain the increased risk.

It should also be pointed out that childhood tumours differ from those on adults regarding their anatomical distribution and histopathology [15]. Moreover, one study on adults that presented results specifically for different types of glioma, e.g., low-grade and high-grade astrocytoma [16], indicated different risk patterns depending on the severity of the disease, with the highest risk being for high-grade astrocytoma. Most astrocytomas in children are of the low-grade type, relatively few being high-grade [15]. In conjunction with the poor prognosis of high-grade astrocytoma (5-year survival of 28% vs. 93% for low-grade), this would indicate that most of the astrocytomas in CEFALO were of the low-grade type. Thus, although the study was relatively small, further subgroup analyses for "astrocytoma and other glioma" would have been of interest (and perhaps other specific tumour types, e.g. ependymoma and medulloblastoma).

Most calculations of laterality show a trend of increasing risk for time since first use, cumulative duration of subscriptions, cumulative duration of calls, and cumulative number of calls (Table five) [11]. However, this trend was seen not only for ipsilateral use, but also for contralateral use. One explanation might be that the preponderance for any particular side in childhood might be less valid than for adults. They use mobile phones in different ways than adults, and might shift the ear depending on their current activity. To disregard the findings without a more thorough analysis is, in this context, not appropriate.

Furthermore, the results in Table five [11] were based on 179 exposed cases versus 194 in total, and 281 exposed controls versus 329 in total. No explanation was given why data was missing for 15 cases and 48 controls compared to the overall analyses. The numbers of "no regular use" for cases and controls also varies in the different calculations. As for the results one might suspect some sort of systematic error, judging by the decreasing trends in risk seen for the category 'central or unknown location', which naturally also casts doubt on the results of the other 2 categories, 'ipsi- and contralateral use', that showed opposite trends, i.e., ORs that increased by exposure. It is difficult to see why the authors chose to combine the centrally located tumours with those of unknown origin. Together these 2 form a larger group of exposed cases (n = 68) than the other 2 (ipsilateral n = 62, contralateral n = 49). With no information on the size of the 'unknown location' group, one cannot form an opinion on whether it is likely to have influenced the results in the categories for ipsi- and contra-lateral use.

### Risk related to cordless phone use

The manner in which the data on cordless phone use were collected and analysed raises additional validity concerns. First, the study should have considered wireless phone use to include both mobile and cordless phones as the exposure category. IARC adopted the term "wireless phone use" as the relevant exposure group [2]. Second, the unexposed group should consist of subjects with no use of wireless phones. Instead, Aydin et al. [11] included use of cordless phones in the 'unexposed' category and thus the risk estimates for mobile phone use might be diluted towards unity. Similarly, mobile phone use was included among the 'unexposed' group when considering use of cordless phones, thereby potentially concealing an increased risk.

For cordless phones, no information on regular use was given, only 'ever use', which was reported by 68.8% (n = 242) of the cases and 66.6% (n = 430) of the controls. Furthermore, use of cordless phones was assessed only 'in the first 3 years' of use, a most peculiar definition for which the authors give neither explanation nor reference. Is there any good evidence to claim that individual differences in use among participants the first 3 years are representative of the following years? If not, this would give rise to exposure misclassification; but even if it was representative, it would not give the complete information regarding cumulative hours of use. In fact, the use of cordless phones increases rapidly over time in childhood; teenagers talk substantially more on cordless phones than children. Thus, excluding use later than the first 3 years probably precludes assessment of a relatively large exposure.

Further, data was provided neither on laterality nor on time since first use. Does the latter mean that all participants had used a cordless phone with a latency time of 3 years? Our studies on use of wireless phones among Swedish children and teenagers (aged 7-19 years conducted during 2005-2006) indicated that the cordless phone was more frequently used for talking than the mobile phone in all age groups [17,18]. In total, 82.9% of the subjects aged 7-19 years reported access to a cordless phone. Of those, 52% talked on average > 5 minutes per day, which was the second lowest exposure category in our study. Talking 5 minutes per day for, e.g., 3 years amounts to ~91 cumulative hours of use, which corresponds to the highest exposure category in CEFALO, > 70 hours. This is equivalent to an average of 3.8 minutes or more per day for 3 years, i.e. in our opinion, not necessarily a highly exposed group. Therefore, it would have been useful to have information regarding the distribution of exposure within that category (n = 25 cases; 38 controls).

Furthermore, numbers in Table six of Aydin et al. [11] are inconsistent. No use of cordless phones overall was reported by 110 cases and 216 controls, but, 102 cases and 189 controls are stated to be unexposed in the calculations of cumulative duration and number of calls. Data are also missing for 116 cases and 224 controls in calculations of the cumulative duration of calls and similar numbers for cumulative number of calls, i.e. ~50% of the cases and the controls reporting "ever use of cordless phones". The authors were careful to note missing cases and controls where mobile phone results were shown (see footnote, Table two) [11], but had no comment why such a large percentage of the data were missing on cordless phones. For both cumulative hours and for cumulative calls the risk seems to increase monotonically with increasing exposure; hence, one wonders what the p-trend value (0.20) in Table six [11] would be had the missing cases been included.

Finally, the authors conclude that no increased brain tumour risk was associated with use of cordless phones in CEFALO and claim that the results contradict estimates of brain tumour risk in our studies that we reported to be of the same order of magnitude for both mobile and cordless phones [16]. Use differs greatly between the studies, which could explain the discrepancy. The highest exposure category for cumulative hours of use in our report was > 2000 hours, while in CEFALO it was > 70 hours.

### Age-dependent risk

The authors compare their results with ours for first use of wireless phone before 20 years of age [3,7]. They neglect the fact that we used wireless phone use as the exposure group (divided into mobile and cordless phones, with no such use as the unexposed category), studied subjects aged 20-80 years, and other tumour types (astrocytoma grade I-IV). Furthermore, the medium latency period for subjects with first use before 20 years of age was 6 years for mobile phone and 7 years for cordless phone, which differs from the much shorter latency period in CEFALO. Their editorial neglected these obvious facts [12].

### Incidence

The authors present incidence data from the Swedish Cancer Registry between 1990-2008 for the age group 5-19 years including hypothetical incidence rate trends, concluding that there was no increase in incidence. Calculations were made on how much the incidence would have increased in the last 10 years (~50%), given a relative risk (OR) of 2.15 after 3 years of regular use of mobile phone (cordless phone use being disregarded). To make the above calculation, information was needed on the proportion of regular use in the source population during these years. That was estimated by combining data from the control subjects in CEFALO with subscriber data in Sweden. First, this calculation implies that 'regular use' (meaning one phone call a week for at least 6 months) is a relevant exposure metric for studying a possible carcinogenic effect of RF-EMF, with which we do not agree (see below). Second, it is not clear how data, as defined in the study, was combined from the controls in CEFALO with subscriber data in Sweden to retrieve the proportion of regular users. Furthermore, we wonder why only data for Sweden was analysed in the time trend analysis. The incidence of brain and central nervous system tumours may not be comparable between Nordic countries because of e.g. different rules regarding the inclusion of benign or unspecified tumours. Based on data in NORDCAN [19], we found in the age group 5-19 years a annual statistically significant increase in incidence of 3.3% (95% CI 0.8 to 5.9) in males and 2.5% (95% CI 0.2 to 4.9) in females for the period 1990-2008 in Norway, a result quite different from Denmark and Sweden (Table 1). Thus, one needs to be cautious using incidence data to dismiss results in analytical epidemiology. It should be noted that the quality of the Swedish Cancer Registry in reporting of CNS tumours, particularly high grade glioma, has been seriously questioned [20,21].

**Table 1:** Estimated change in incidence rate/year (%) and 95% confidence interval (CI) for brain and central nervous system tumours in the age group 5-19 years in Nordic countries 1990-2008 according to NORDCAN

	Men			Women		
	**Change in incidence rate/year (%)**	**95% CI**	**n**	**Change in incidence rate/year (%)**	**95% CI**	**n**

Nordic countries*						
1990-2008	-0.1	-1.2, 1.1	1 779	+0.8	-0.3, 2.0	1 541

Denmark						
1990-2008	-1.1	-3.5, 1.3	418	+0.5	-1.9, 3.1	347

Norway						
1990-2008	+3.3	0.8, 5.9	347	+2.5	0.2, 4.9	314

Sweden						
1990-2008	-2.3	-3.5, -1.1	631	+0.3	-1.6, 2.2	546

Calculations were based on incidence rates age adjusted to the world standard population and rounded to two decimal places. Linear regression analysis on the logarithm of the age-adjusted incidence rates was used to calculate the trends.

* = Denmark, Finland, Iceland, Norway, Sweden.

## Conclusions

Further studies are required on the risk of brain tumours in children associated with use of mobile and cordless phones; future investigations must meet basic demands on quality, by which we mean not only efforts undertaken to assure reliable assessment of exposure and outcome, but also to obtain a sufficient number of cases and controls of which not just a few by modern measures have been exposed. The biological mechanism by which RF-EMF exposure might cause or promote cancer remains unknown. Since we lack that knowledge, any assumption made about exposure-response relations and the threshold of any increased risk is premature. In view of the latter and the limited statistical power in CEFALO the main conclusion of Aydin et al. of lack of an exposure-response relationship argues against a causal association seems unwarranted.

Our own research shows that some teenagers, particularly girls, report spending several hours on average per day using a cordless phone. If that information is correct, their amount of cumulative use may exceed 70 hours in a month, not years like that reported in CEFALO. The study was relatively small and scarcely gives any information on the risk for heavy and long-term use, and certainly is not comparable to studies on adults where notably an increased risk of glioma has consistently been shown for longer latency times, e.g. > 7 [22] and 10 years [3-7]. Yet, in spite of low exposure, short latency period and limitations in study design, analyses and interpretation, there are nevertheless indications of increased risk in CEFALO. In any case, it is to go far beyond the findings of the study to say that the results are reassuring of no significant increased risk.

Finally, carcinogenesis is a multistage event and the cancer incidence depends on initiation, promotion and progression of the disease [23]. It follows that, since the mechanism for a possible carcinogenic effect of RF-EMF exposure is unknown, descriptive incidence data are of limited value and should currently be of secondary importance to those based on analytical epidemiology.

## List of abbreviations

CI: Confidence interval; DECT: Digital Enhanced Cordless Telecommunication; IARC: International Agency for Research on Cancer; OR: Odds ratio; RF-EMF: Radiofrequency Electromagnetic Fields.

## Competing interests

The authors declare that they have no competing interests.

## Authors' contributions

FS, MC and LH shared efforts to scrutinize the literature in question. KHM also gave his comments. FS were responsible for drafting the manuscripts under advice and significant input from MC, LH. KHM read and gave his comments of the manuscript. All authors have read and approved the final version of the paper before submission.

## Endnotes

A. A letter was submitted to JNCI soon after Aydin et al. published their results. Two months later we had received no decision (or reply) in the matter why we decided to withdraw the letter and instead write this commentary.

B. Due to the lack of a national population registry in Switzerland a two-stage sampling procedure was applied: first, by randomly determining a community within the same language region as the patient, and second, by randomly selecting a control subject from the corresponding communal population registry.
